# Screening and evaluation of lactic acid bacteria with probiotic potential from local Holstein raw milk

**DOI:** 10.3389/fmicb.2022.918774

**Published:** 2022-08-01

**Authors:** Wenqing Zhang, Shiji Lai, Ziyao Zhou, Jinpeng Yang, Haifeng Liu, Zhijun Zhong, Hualin Fu, Zhihua Ren, Liuhong Shen, Suizhong Cao, Lei Deng, Guangneng Peng

**Affiliations:** ^1^Key Laboratory of Animal Disease and Human Health of Sichuan Province, College of Veterinary Medicine, Sichuan Agricultural University, Chengdu, China; ^2^Laboratory of Molecular and Cellular Parasitology, Department of Microbiology and Immunology, Healthy Longevity Translational Research Programme, Yong Loo Lin School of Medicine, National University of Singapore, Singapore, Singapore

**Keywords:** raw milk, LAB strains, probiotic potential, safety assessment, probiotics, *Lactiplantibacillus plantarum*

## Abstract

There are massive bacteria in the raw milk, especially the lactic acid bacteria (LABs), which have been considered probiotics in humans and animals for a long time. Novel probiotics are still urgently needed because of the rapid development of the probiotic industry. To obtain new LABs with high probiotic potential, we obtained 26 LAB isolates, named L1 ~ L26, from local Holstein raw milk collected from a farm whose milk had never been used for LAB isolation. We identified them at the species level by biochemical and 16S rDNA sequencing methods. Their antagonistic activities against four target pathogens (*Escherichia coli* ATCC 25922, *Staphylococcus aureus* ATCC 25923, *Pseudomonas aeruginosa* PAO1, and *Salmonella enterica* H9812), co-aggregative ability with these target pathogens, survivability in the simulated gastrointestinal tract conditions and phenol, auto-aggregation and hydrophobicity, hemolytic activity, and antibiotic susceptibility, were evaluated *in vitro*. Five *Lactiplantibacillus plantarum* isolates (L5, L14, L17, L19, and L20) showed more promising probiotic potential than others. Specifically, these five isolates conglutinated with and inhibited all the target pathogens, and survived in the simulated gastric juice (92.55 ~ 99.69%), intestinal juice (76.18 ~ 83.39%), and 0.4% phenol (76.95 ~ 88.91%); possessed considerable auto-aggregation (83.91 ~ 90.33% at 24 h) and hydrophobicity (79.32 ~ 92.70%); and were non-hemolytic, sensitive to kinds of common antimicrobials. Our findings demonstrated that these five isolates could be preliminarily determined as probiotic candidates because they have better probiotic potential than those previously reported. Again, this study highlighted the potential of raw milk for probiotic isolating and screening and provided the probiotic industry with five new LAB candidates.

## Introduction

Probiotics are defined as a kind of “live microorganisms which when administered in adequate amounts confer a health benefit on the consumer” (Araya et al., 2002). Lactic acid bacteria (LABs), including *Lactococcus*, *Lactobacillus*, *Streptococcus*, and *Enterococcus*, are a group of Gram-positive, cocci or rod, catalase-negative, coagulase-negative, non-spore-forming, and harsh to culture bacteria. LABs possess a high tolerance for low pH (Mokoena, 2017; De Melo Pereira et al., 2018) and have been considered one of the most important probiotics for a long history because of their extensive beneficial effects on humans and animals (Food and Agriculture Organization, 2006; Hill et al., 2014). It has been determined that LABs possess anti-cancer, anti-cholesterol, anti-depression, anti-anxiety, anti-obesity, anti-diabetic, and immunostimulatory activities (Zoumpopoulou et al., 2017; Suez et al., 2019; Mathur et al., 2020). Intriguing, LABs are also considered a role in the respiratory system (De Boeck et al., 2021). The LABs-sourced biofunctional products are still in great need even though diverse functional LABs have been applied in commercial probiotic fermented food worldwide (De Melo Pereira et al., 2018). An approximate 27.9 billion dollars were spent on the purchase of probiotics in 2011, which increased to 44.9 billion dollars in 2018 (Transparency Market Research (TMR), 2013). And the global probiotics demand was expected to increase to 83.5 billion dollars by 2022 T.T.M. Research (2017). Meanwhile, abundant scientific studies involving the selection of LABs with different and specific functional properties have been reported in the last decades, and new probiotic LABs are also being constantly isolated and identified (De Melo Pereira et al., 2018). These new LABs are isolated from multiple sources, such as human raw milk (Shin et al., 2021) and grains (Fiorda et al., 2017), but milk and other dairy products are commonly considered the main sources of LABs (Plessas et al., 2017; Reuben et al., 2020).

Considering the target functions and technological applications, screening, selecting, and evaluating new probiotic LABs require a comprehensive approach consisting of a series of steps (De Melo Pereira et al., 2018). In 2002, the Food and Agriculture Organization of the United Nations and the World Health Organization published the “Guidelines for Evaluation of Probiotics in Food” (Araya et al., 2002), which put an end to the chaos in affirming the efficacy and safety of probiotic microorganisms, and established safety and effectiveness standards for probiotics selection and evaluation. The guidelines proposed several criteria for the selection of probiotics. Firstly, the candidates should possess the ability against the unfavorable conditions imposed by the human body, including the enzymes, adverse pH, mild heat shock, bile acid, phenol, etc. Secondly, the candidates should also possess the ability to colonize the gastrointestinal tract (GIT) epithelial cells, called adhesion ability, consisting of both autoaggregation capacity and hydrophobic properties. Once adhered to the epithelial cells, the candidates should produce extracellular antimicrobial ability by converting carbohydrates, proteins, and other minor compounds into important substances that can inhibit pathogenic bacteria or by competing for nutrients, aggregating with pathogens, and stimulating the immune system (Lebeer et al., 2008). Furthermore, the safety must be assessed when live microbes are introduced to the daily diet (Culligan et al., 2009), including isolation history, taxonomic identification, absence of virulence, infectivity, toxicity, and transferable antibiotic resistance genes (Sanders et al., 2010). Finally, after *in vitro* studies, animal studies and clinical trials should also be executed to validate the safety and efficiency of the final candidates (De Melo Pereira et al., 2018).

Hence, to obtain new LABs with promising probiotic potential, we collected cow milk samples from indigenous Holstein cows raised on a historic farm whose milk had never been sampled for probiotics isolation and isolated and identified the LABs in the milk. Then, the antipathogenic activity, stress tolerance, adhesion activity, safety characteristics, and growth performance were assessed. Our study will provide new probiotic LAB candidates for further probiotic development and industry.

## Materials and methods

### Sample collection

Fresh milk samples were randomly collected from 22 healthy (puerperal period, 524 ± 58 kg, without visible symptoms) Holstein cows belonging to a large-scale farm (over 1,000 puerperal cows, 29.91°N, 103.37°E) with over 50 years of history in Hongya, Sichuan, China. Before the sample collection, the udder and the surrounding area were thoroughly cleaned with 70% ethanol and dried with individual paper towels. The sample was collected into 50 ml sterile corked plastic tubes after discarding the first three drops of raw milk, followed by immediate storage in a 4°C ice-box, transportation to the laboratory for the following experiments.

### Isolation and purification

The isolation method of LABs referred to Reuben et al. (2020). Briefly, 10 ml of each milk sample was enriched with 40 ml of de Man, Rogosa, and Sharpe (MRS) broth (Hopebio, Qingdao, China) and cultured overnight in a 37°C-shaking incubator under aerobic conditions. The fresh cultures with visible turbidity were homogenized in sterile normal saline using a vortex mixer, and 100 μl of each sample after ten-fold of continuous dilution was taken and coated on MRS agar and incubated for 24–72 h at 37°C under aerobic conditions. Then, individual colonies with different morphologies were selected and purified through three continuous passages on MRS agar. According to the standard procedures (Sharpe, 1979), catalase activities and coagulase activities were detected. Together with the Gram staining results (positive), catalase activity (negative), coagulase activity (negative), and cell morphology, 26 purified isolations (with different colony characteristics) were preliminarily identified as LABs and stored at −80°C in 50% glycerol for the subsequent experiments.

### Species identification

#### Biochemical identification

The biochemical characteristics of the 26 purified LAB isolates were simultaneously identified using biochemical tubes (Hopebio, Qingdao, China) according to Bergey’s Manual of Determinative Bacteriology (Cummings, 1926), including the ability to ferment different sugars, gelatin liquefaction, and sulfuretted hydrogen production.

#### 16S rDNA sequencing and sequences analysis

The DNA of the 26 LAB isolates was extracted by a DNA Extraction Kit (Tiangen, Beijing, China), and the quality of the extracted DNA was measured using an ND-1000 micro UV spectrophotometer (NanoDrop Technologies, United States). Then, the universal primer 27F (5′-AGAGTTTGATCCTGGCTCAG-3′) and 1492R (5′-TACGACTTAACCCCAATCGC-3′) were used to amplify the 16S rDNA gene. Each PCR reaction (25 μl) contained 12.5 μl PCR Master Mix, 9.5 μl nuclease-free H_2_O, 1 μl forward primer, 1 μl reverse primer, and 1 μl DNA sample. The PCR procedure was performed as follows: predenaturation at 94°C for 5 min, followed by 30 cycles (30 s of denaturation at 94°C, 30 s of annealing at 55°C, and 1 min of extension at 72°C), with a final extension at 72°C for 7 min. The PCR products were stored at 4°C for subsequently checking on 2% agarose gel electrophoresis stained with the golden view. Part of the checked products was then sent to Sangon Biotech Co.Ltd. (Shanghai, China) for 16S rDNA sequencing. Based on the results of 16S rRNA sequencing, the homology alignment analysis with the nucleic acid sequences of bacteria in GenBank^1^ was performed using BLAST.^2^ Then, the phylogenetic tree was established by MEGA6 software (Mega Limited, Auckland, New Zealand) and sequences with a demarcation threshold of >99% were classified as the same species. Kimura 2-parameter model and Neighbor-Joining method were used to construct the phylogenetic tree. Briefly, the robustness of individual branches was estimated using bootstrapping with 1,000 replications, and the phylogenetic tree was confirmed by the maximum-parsimony method and maximum-likelihood method. *Lactococcus lactis* strain NBRC 100933 (NR_113960.1), *L. lactis* strain 4,319 (MT544861.1), *Streptococcus lutetiensis* strain CIP 106849 (NR_115719.1), *Weissella hellenica* strain NCFB 2973 (NR_118771.1), *Enterococcus durans* strain 98D (NR_036922.1), *Limosilactobacillus fermentum* strain NBRC 15885 (NR_113335.1), *Limosilactobacillus fermentum* strain CIP 102980 (NR_104927.1), *Enterococcus lactis* strain BT159 (NR_117562.1), *Rummeliibacillus stabekisii* strain KSC-SF6g (NR_043992.1), *Lactobacillus plantarum* strain DKO 22 (NR_042254.1), and *L. plantarum* strain DSM 10667 (NR_025447.1) were used as typical strains to construct the phylogenetic tree.

### Antipathogenic activity detection

#### Antagonistic activity

The antimicrobial activities of the 26 LAB isolates against enterotoxigenic *Escherichia coli* ATCC 25922 (ETEC), *Staphylococcus aureus* ATCC 25923, *Pseudomonas aeruginosa* PAO1, and *Salmonella enterica* H9812 were determined using the Oxford Cup method (Fontana et al., 2015). These four pathogenic bacteria were purchased from the American type culture collection (ATCC). Briefly, the resurgent LAB isolates were inoculated to MRS broth and incubated for 24 h at 37°C under aerobic conditions. Meanwhile, the targeted pathogens were precultured under the same conditions in Luria-Bertani (LB) broth (Hopebio, Qingdao, China). Fresh cultures of the four targeted pathogens (100 μl, 10^7^ CFU/ml) were coated on an LB agar plate and dried. Oxford Cups placed on plates were filled with 100 μl of cell-free supernatant (CFS) obtained from centrifugation of LAB cultures at 4500 r/min for 10 min. The diameters of inhibition zones were measured and recorded after incubating at 37°C for 24 h under anaerobic conditions.

#### Co-aggregative ability with pathogens

The co-aggregation abilities of 14 LAB isolates (with inhibitory effect on all four target pathogens) were detected to evaluate their abilities to gather pathogens and facilitate the elimination of pathogens through feces (De Melo Pereira et al., 2018). Briefly, 2 ml of fresh overnight cultures and 2 ml of each pathogen culture were mixed, vortexed, and incubated at 37°C for 2 h. Tubes containing 4 ml of each LAB isolates or each pathogen suspension were used as controls. Then, the absorbance (600 nm) at 2 h of the tubes was measured to calculate the co-agglutination rates followed the formula:


$$\begin{array}{c} \mathrm{co}-\mathrm{agglutination rate}\left(\%\right)=1-A_{\mathrm{mix}}/\left[\left(A_{\mathrm{LAB}}+A_{\mathrm{pathogen}}\right)/2\right]\\ \times 100 \end{array}$$

in which A_mix_ represents the absorbance of the mixture, A_LAB_ represents the absorbance of the pure LAB cultures, and A_pathogen_ represents the absorbance of the pure pathogen suspension.

### Stress tolerance detection

#### Tolerance for simulated GIT conditions

The survivability of the 14 selected LAB isolates to simulated GIT conditions was assessed referred to Zhang’s work (Zhang et al., 2016). Firstly, 0.3 g pepsin (Solarbio, Beijing, China) was dissolved in 100 ml 0.9% sterile saline, and the pH was adjusted to 3.0 with 1 M HCL (Hopebio, Qingdao, China) to prepare the simulated gastric juice. And 0.2 g trypsin (Sangon, Beijing, China) and 0.3 g ox-bile salts (Hopebio, Qingdao, China) were dissolved in 100 ml 0.9% sterile saline, and the pH was adjusted to 8.0 with 1 M NaOH (Hopebio, Qingdao, China) to prepare the simulated intestinal juice. The simulated gastric juice and intestinal juice were subsequently filter-sterilized (0.22 μm; Green Union Science Instrument Co., Ltd, Jiangsu, China). After three consecutive passages, the resurgent LAB isolates were incubated in MRS broth for 12 h. Then, 10 ml of the fresh cultures were centrifuged at 8,000 × g for 10 min at 25°C. The pelleted cells were resuspended in an equal volume of sterile normal saline, followed by 10 min of centrifugation with the same parameters. Then, the pelleted cells were resuspended in 10 ml of prepared simulated gastric juice (0 h), followed by incubation at 37°C for 3 h under aerobic conditions (3 h). After that, the pelleted cells obtained by centrifugation from gastric juice were transferred into 10 ml of prepared simulated intestinal juice again and incubated aerobically for 4 h at 37°C (7 h). The viable colonies at 0 h, 3 h, and 7 h were determined using plate counts on MRS agar to calculate the survival rate. All the experiments were repeated three times with three technical replicates each time. The mean value of the results of the three independent experiments was calculated as follows:


$$Survivalrate\;\left(\%\right)=N_{1}/N_{0}\times 100,$$

in which *N*_0_ is the number of viable bacteria at 0 h (CFU/mL) and *N*_1_ is the number of viable bacteria in artificial gastrointestinal fluid at 3 or 7 h (CFU/mL).

#### Tolerance for phenol

To assess the phenol tolerance of the 14 selected LAB isolates, overnight LAB cultures were transferred to a new MRS broth containing 0.4% phenol (Hopebio, Qingdao, China) at 37°C. After 24 h of incubation, the viable colonies of cultures were measured using plate counts to detect the viability of the LAB isolates.

### Adhesion activity detection

#### Auto-aggregation activity

The auto-aggregation abilities of the 14 selected LAB isolates were determined using the spectrophotometer to evaluate the adherence capability to intestinal epithelial cells. Briefly, fresh cultures were centrifuged at 4500 r/min for 10 min to collect the pelleted cells, washed twice with sterile 1 × PBS, and adjusted to 10^8^ CFU/ml in the same buffer. Then, 4 ml of the adjusted cell suspension was vortexed for 10 s and incubated for 24 h at 37°C. To observe the auto-aggregation ability, the absorbance (600 nm) at 0, 3, 6, and 24 h was measured using a spectrophotometer. All the experiments were repeated three times with three technical replicates each time. The mean value of the results of the three independent experiments was calculated as follows:


$$auto-agglutinationrate\;\left(\%\right)=1-\left(A_{t}/A_{0}\right)\times 100,$$

in which *A*_t_ represents the absorbance at 3, 6, or 24 h and *A*_0_ represents the absorbance at 0 h.

#### Cell surface hydrophobicity

To evaluate the adherence ability to hydrocarbons of the selected LAB isolates, the cell surface hydrophobicity was measured. Firstly, fresh overnight LAB cultures were collected by centrifugation at 4,500 r/min for 10 min, and the pelleted cells were washed twice with sterile 1 × PBS and then resuspended in the same buffer. Afterward, 2 ml of cell suspension was mixed with 2 ml xylene (Sinopharm Chemical Reagent Co., Ltd., Shanghai, China), and the mixtures were vortexed vibration for 10 min and left at 25°C for 40 min for two phases separation. The lower aqueous phase was carefully absorbed, and its absorbance was measured at 600 nm in triplicate to calculate the cell surface hydrophobicity (%), the formula is as follows:


$$Cellsurfacehydrophobicity\;\left(\%\right)=1-\left(A_{f}/A_{0}\right)\times 100$$

in which *A*_f_ represents final absorbance and *A*_0_ represents initial absorbance.

### Safety assessment

#### Hemolytic activity

To determine the hemolytic activity, fresh overnight LAB cultures were streaked on blood agar plates, and the phenotype around the colonies was observed after 48 h of incubation at 37°C. *S. aureus* ATCC 25923 was used as the positive control. The hemolytic reaction was evaluated by observing both the partial hydrolysis of red blood cells and the production of a green zone (α-hemolysis), as well as the total hydrolysis of red blood cells producing a clear zone around the bacterial colony (β-hemolysis) or no reaction (γ-hemolysis).

#### Antibiotic susceptibility

Disc-diffusion test was used to assess the antibiotic susceptibility of the selected LAB strains, including the following 13 antimicrobials (Lanjun Biotechnology Co., Ltd., Guangzhou, China): penicillin G (P, 10 μg), ampicillin (AMP, 10 μg), ceftriaxone (CRQ, 30 μg), amoxicillin (AML, 25 μg), erythromycin (E, 15 μg), clarithromycin (CLR, 15 μg), tetracycline (TE, 30 μg), gentamicin (C, 10 μg), amikacin (AK, 30 μg), vancomycin (VA, 30 μg), chloramphenicol (C, 30 μg), rifampicin (Rd, 5 μg), and fosfomycin (S, 200 μg). Fresh overnight cultures of each LAB strain were diluted to 10^8^ CFU/ml, 100 μl of which was coated on MRS agar plates and dried. Then, three homogenous antibiotic discs were manually placed on the surface of the dried MRS plate. After 5 min, the placed plates were turned over and incubated at 37°C for 48 h under anaerobic conditions. The diameters (mm) of the inhibition zones were measured to classify the antibiotic susceptibility as resistance (R), moderate susceptibility (MS), or susceptibility (S) based on the parameters of the Clinical and Laboratory Standards Institute (CLSI; El-Shaer et al., 2017).

### Growth performance evaluation

Referring to the previous study (Liu et al., 2020), we detected the growth performance of the five candidates by constructing their growth curves using MRS broth as the negative control. Briefly, 50 μl (1%) of each LAB culture (the mid-exponential phase) was inoculated into 50 ml of fresh MRS broth and incubated at 37°C for 48 h. The absorbance (600 nm) was measured at a frequency of every 2 h in the 0–24 h and every 6 h in the 25–48 h.

### Statistical analysis

All results were expressed as mean ± SD, and the statistical significance of the differences was evaluated by one-way ANOVA using SPSS 26 (IBM, NYC, United States). Differences were considered significant at *p* < 0.05 and extremely significant at *p* < 0.01. All the graphical presentations were generated by GraphPad Prism 9.0 (GraphPad Software, CA, United States).

## Results

To obtain different LABs, we picked out individual colonies with different morphologies. And a total of 26 isolates with typical morphological characteristics of LAB (Gram-positive *bacilli* and *cocci*, catalase-negative, coagulase-negative, and non-motile) were obtained from the 22 cow milk samples after isolation and purification for subsequent experiments, including species identification, antipathogenic activity detection, stress tolerance detection, adhesion activity detection, safety assessment, and growth performance evaluation.

### Species identification

**Figure 1:**
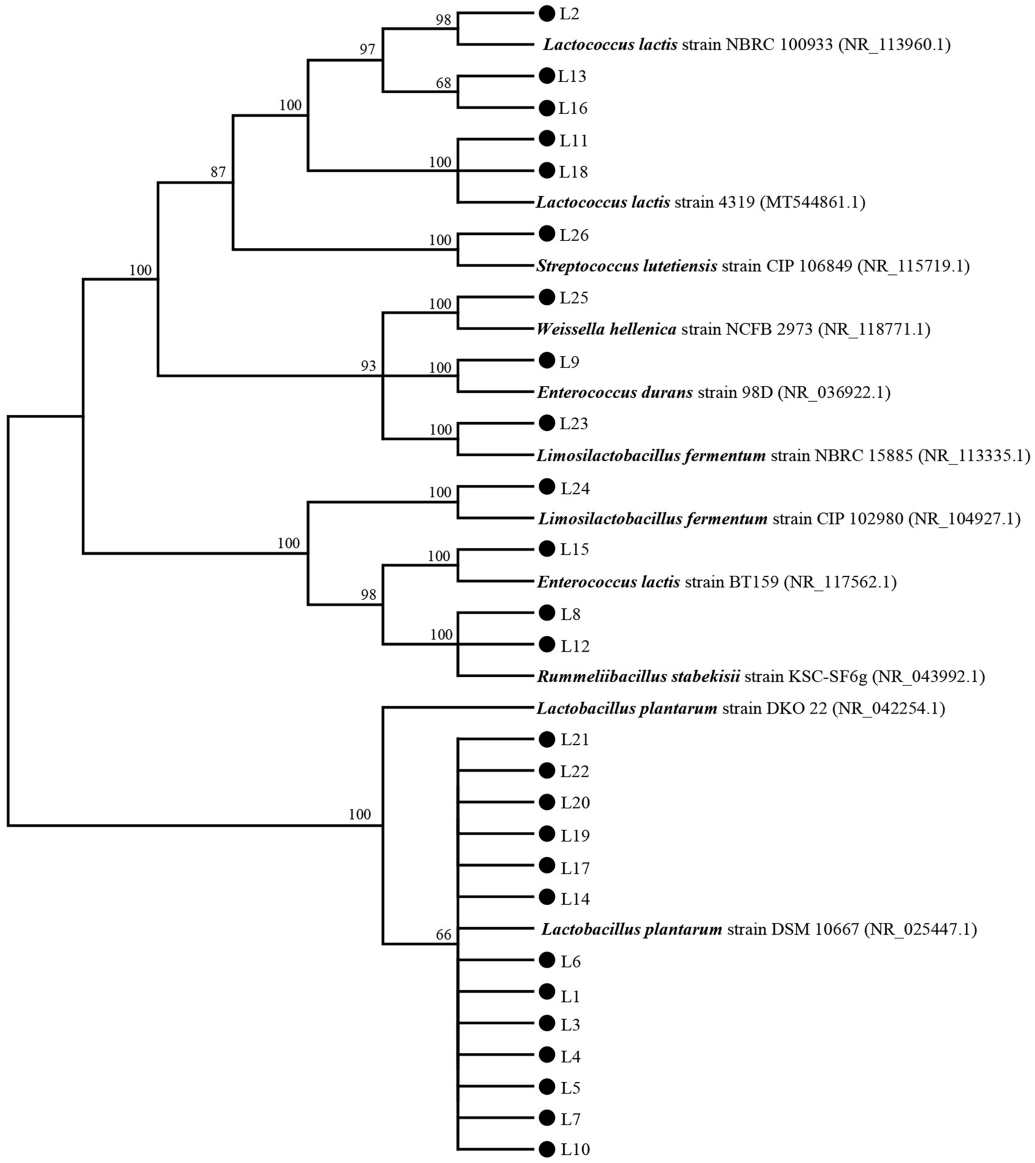
The phylogenetic tree based on 16S rDNA genes of the 26 isolates and type strains.

The biochemical characteristics of the 26 isolates were detected to confirm the type of these isolates preliminarily. The results are shown in Supplementary Table 1. Based on the biochemical characteristics, the 26 isolates were initially identified as *Lactococcus* (L2, L11, L13, L16, and L18), *Streptococcus* (L26), *Lactiplantibacillus* (L1, L3, L4, L5, L6, L7, L10, L14, L17, L19, L20, L21, and L22), *Enterococcus* (L9 and L15), *Rummeliibacillus* (L8 and L12), *Limosilactobacillus* (L23 and L24), and *Weissella* (L25). The 16S rDNA gene sequences of these 26 isolates were used to construct a phylogenetic tree, and the result is shown in Figure 1. L2, L13, and L16 were clustered with *Lactococcus lactis* strain NBRC 100933 (NR_113960.1); L11 and L18 were clustered with *L. lactis* strain 4,319 (MT544861.1); L1, L3, L4, L5, L6, L7, L10, L14, L17, L19, L20, L21, and L22 were clustered with *Lactiplantibacillus plantarum* strain DSM 10667 (NR_025447.1); L23 were clustered with *Limosilactobacillus fermentum* strain NBRC 15885 (NR_113335.1); L24 were clustered with *Limosilactobacillus fermentum* strain CIP 102980 (NR_104927.1); L25 were clustered with *W. hellenica* strain NCFB 2973 (NR_118771.1); L26 were clustered with *S. lutetiensis* strain CIP 106849 (NR_115719.1); L8 and L12 were clustered with *R. stabekisii* strain KSC-SF6g (NR_043992.1); and L15 and L9 were clustered with *Enterococcus lactis* strain BT159 (NR_117562.1) and *E. durans* strain 98D (NR_036922.1), respectively.

### Antipathogenic activity

To evaluate the inhibitory effect of the obtained 26 isolates on the growth of common intestinal pathogens, the antagonistic activities against *E. coli* ATCC 25922 (ETEC), *S. aureus* ATCC 25923, *P. aeruginosa* PAO1, and *S. enterica* H9812 were detected. We observed that 14 of them inhibited the growth of all 4 pathogens. The detailed results of the antipathogenic activities are shown in Table 1. Referring previous study (Reuben et al., 2020), the antipathogenic activities of these LABs were divided into four ranges: I, 8 mm < zone diameters ≤12 mm; II, 12 mm < zone diameters ≤16 mm; III, 16 mm < zone diameters ≤20 mm; and IV, 20 mm < zone diameters. In general, these 14 isolates demonstrated high antagonistic activities against *S. aureus* ATCC 25923, moderate antagonistic activities against *P. Aeruginosa* PAO1, and low antagonistic activities against ETEC and *Salmonella* H9812 (*p* < 0.05). Although the inhibitory effect on the 4 target pathogens was strain-specific, the isolates L17, L1, L21, L14, and L19 exhibited higher inhibitory activities than others (*p* < 0.05).

**Table 1:** Antagonistic activity of potential probiotic isolates from cow milk samples against four target pathogenic bacteria by the Oxford cup method.^1^

Strain	Antagonistic activity (mm)			
	*E. coli* ATCC 25922	*Staphylococcus aureus* ATCC 25923	*Salmonella* H9812	*P. aeruginosa* PAO1
L1	14.57 ± 0.45^bc^	27.43 ± 0.51^a^	12.57 ± 0.46^c^	18.40 ± 0.29^b^
L2	11.10 ± 0.26^e^	12.43 ± 0.42^i^	13.60 ± 0.15^b^	14.27 ± 0.20^i^
L3	14.20 ± 0.61^cd^	22.13 ± 0.51^b^	11.07 ± 0.12^fg^	15.73 ± 0.25^fgh^
L4	14.30 ± 0.78^bcd^	21.93 ± 0.72^bc^	10.77 ± 0.26^g^	16.93 ± 0.15^de^
L5	14.50 ± 0.89^bc^	21.93 ± 0.50^bc^	12.70 ± 0.25^c^	18.07 ± 0.87^bc^
L6	13.17 ± 0.42^d^	21.27 ± 0.90^cd^	11.10 ± 0.60^fg^	15.63 ± 0.46^gh^
L7	13.53 ± 1.04^de^	14.40 ± 0.10^h^	14.30 ± 0.10^a^	16.10 ± 0.70^fg^
L10	14.03 ± 0.25^cd^	27.43 ± 0.45^a^	11.93 ± 0.21^de^	15.03 ± 0.15^ghi^
L14	12.90 ± 0.82^d^	20.97 ± 0.49^d^	12.40 ± 0.32^cd^	16.13 ± 0.26^hi^
L17	13.97 ± 0.45^cd^	20.13 ± 0.93^e^	14.43 ± 0.50^a^	22.03 ± 0.55^a^
L19	16.53 ± 0.55^a^	19.53 ± 0.64^ef^	12.13 ± 0.36^cde^	16.50 ± 0.15^ef^
L20	14.53 ± 0.21^bc^	19.23 ± 0.21^f^	11.60 ± 0.15^ef^	12.63 ± 0.30^j^
L21	15.07 ± 0.15^b^	26.87 ± 0.25^a^	11.57 ± 0.64^ef^	17.47 ± 0.35^cd^
L22	16.43 ± 0.75^a^	18.10 ± 0.36^g^	11.60 ± 0.26^ef^	15.60 ± 0.17^gh^

1 *Results of independent experiments (n = 3) of inhibition zones are presented using mean ± SD;*

^a-j^Values in a column with different superscript letters are significantly different (Waller-Duncan, *p* < 0.05).

The co-aggregation abilities of the 14 isolates with the four target pathogens are shown in Table 2. For ETEC, L20 showed the highest co-aggregation ability, followed by L5, L14, L7, and L22. For *S. aureus* ATCC 25923, the isolates with the top five highest co-aggregation abilities were L5, L20, L22, L14, and L7. For *Salmonella* H9812, L22 showed the highest co-aggregation ability, followed by L5, L20, and L14. For *P. Aeruginosa* PAO1, the isolates with the top five highest co-aggregation abilities were L22, L20, L14, L7, and L10.

**Table 2:** Co-aggregation abilities of potential probiotic LABs isolated from cow milk.^1^

Strain	Co-aggregation (%)			
	*E. coli* ATCC 25922	*Staphylococcus aureus* ATCC 25923	*Salmonella* H9812	*P. aeruginosa* PAO1
L1	35.96 ± 0.64^gh^	31.07 ± 0.95^i^	30.92 ± 1.22^k^	53.49 ± 1.16^gh^
L2	38.42 ± 1.53^ef^	34.95 ± 0.58^g^	36.05 ± 3.15^j^	53.40 ± 0.52^h^
L3	28.43 ± 1.33^i^	29.91 ± 1.14^j^	41.97 ± 1.33^i^	54.66 ± 0.52^fg^
L4	36.71 ± 1.67^fg^	41.63 ± 1.01^e^	53.70 ± 1.59^g^	47.26 ± 0.42^i^
L5	66.51 ± 1.01^ab^	74.24 ± 0.37^a^	94.37 ± 2.12^b^	83.48 ± 1.15^b^
L6	34.30 ± 0.82^h^	29.12 ± 0.67^j^	48.37 ± 2.35^h^	46.97 ± 0.31^i^
L7	52.99 ± 1.37^c^	50.49 ± 1.17^d^	63.19 ± 1.74^e^	65.15 ± 1.02^d^
L10	38.74 ± 0.81^e^	35.26 ± 0.83^g^	52.04 ± 2.18^g^	58.14 ± 1.63^e^
L14	65.45 ± 1.66^b^	56.43 ± 0.22^c^	84.91 ± 2.16^d^	81.00 ± 0.69^c^
L17	50.65 ± 1.46^d^	33.33 ± 0.24^h^	46.19 ± 1.20^h^	54.83 ± 1.06^f^
L19	49.61 ± 1.63^d^	37.16 ± 0.92^f^	57.25 ± 0.92^f^	55.31 ± 1.09^f^
L20	67.85 ± 1.10^a^	73.18 ± 0.48^a^	90.75 ± 1.03^c^	82.99 ± 0.19^b^
L21	21.63 ± 0.92^j^	34.60 ± 0.34^g^	27.28 ± 1.38^m^	37.09 ± 0.15^j^
L22	52.95 ± 0.41^c^	69.68 ± 0.71^b^	97.19 ± 1.04^a^	87.41 ± 0.03^a^

1 *Results of independent experiments (n = 3) of co-aggregation rate are presented using mean ± SD;*

^a-m^Values in a column with different superscript letters are significantly different (Waller-Duncan, *p* < 0.05).

### Tolerance for simulated GIT conditions and phenol

In order to detect the survival ability of the isolated LABs in a simulated gastrointestinal environment, these 14 isolates were inoculated in artificial gastric juice for 3 h, then transferred to the artificial intestinal juice for 4 h. The results are shown in Table 3. In general, all the isolates showed high survival rates (74.49 ~ 99.69%) in the simulated gastric juice, and most of the isolates (except L2) demonstrated high survival rates (64.95 ~ 84.93%) in the simulated intestinal juice. Specifically, the five isolates with the highest survival rate in gastric juice were L22 (99.69%), L21 (96.92%), L7 (98.05%), L3 (97.82%), and L19 (96.37%), and L21 (84.94%), L22 (83.89%), L14 (83.39%), L4 (82.60%), and L17 (80.48%) were the top five isolates with the highest survival rates in the artificial intestinal juice.

**Table 3:** Survival of the potential probiotic isolates in the artificial gastric and intestinal juices.^1^

Strain	Initial concentration	Artificial gastric juice at pH 3.0		Artificial intestinal juice at pH 8.0	
	0 h (log10 CFU ml^−1^)	3 h (log10 CFU ml^−1^)	Survival rate (%)	7 h (log10 CFU ml^−1^)	Survival rate (%)
L1	9.71 ± 0.01^a^	7.23 ± 0.10^f^	74.49 ± 1.04^f^	7.31 ± 0.08^e^	75.25 ± 0.85^g^
L2	8.90 ± 0.01^gh^	7.85 ± 0.06^e^	88.20 ± 0.66^e^	3.00 ± 0.00^j^	33.72 ± 0.00^m^
L3	8.88 ± 0.05^h^	8.68 ± 0.06^bc^	97.82 ± 0.63^ab^	6.32 ± 0.03^h^	71.19 ± 0.40^h^
L4	9.41 ± 0.05^cd^	8.05 ± 0.06^de^	90.91 ± 0.44^de^	7.77 ± 0.06^b^	82.60 ± 0.66^c^
L5	9.47 ± 0.09^c^	8.77 ± 0.56^b^	92.55 ± 5.86^cd^	7.22 ± 0.02^f^	76.18 ± 0.13^f^
L6	9.34 ± 0.03^de^	8.88 ± 0.08^b^	95.09 ± 0.82^bc^	6.07 ± 0.02^i^	64.96 ± 0.24^k^
L7	9.58 ± 0.06^b^	9.40 ± 0.06^a^	98.05 ± 0.55^ab^	7.40 ± 0.03^d^	77.20 ± 0.31^e^
L10	8.97 ± 0.06^g^	8.72 ± 0.08^bc^	97.28 ± 0.80^ab^	6.00 ± 0.00^i^	66.91 ± 0.00^j^
L14	9.34 ± 0.03^de^	8.88 ± 0.03^b^	95.05 ± 0.34^bc^	7.79 ± 0.07^b^	83.39 ± 0.74^bc^
L17	9.35 ± 0.05^de^	8.98 ± 0.09^b^	96.03 ± 0.95^b^	7.53 ± 0.08^c^	80.48 ± 0.88^d^
L19	9.29 ± 0.01^e^	8.95 ± 0.04^b^	96.37 ± 0.42^b^	6.99 ± 0.09^g^	75.25 ± 0.95^g^
L20	9.14 ± 0.05^f^	8.37 ± 0.06^cd^	91.59 ± 0.67^d^	6.24 ± 0.03^h^	68.22 ± 0.32^i^
L21	9.57 ± 0.03^b^	8.93 ± 0.05^b^	96.92 ± 0.49^ab^	8.12 ± 0.01^a^	84.94 ± 0.16^a^
L22	9.64 ± 0.05^ab^	9.60 ± 0.06^a^	99.69 ± 0.62^a^	8.08 ± 0.03^a^	83.89 ± 0.22^b^

1 *Results are expressed as mean ± SD;*

*^a-m^Values in a column with different superscript letters are significantly different (Waller-Duncan, p < 0.05)*.

**Figure 2:**
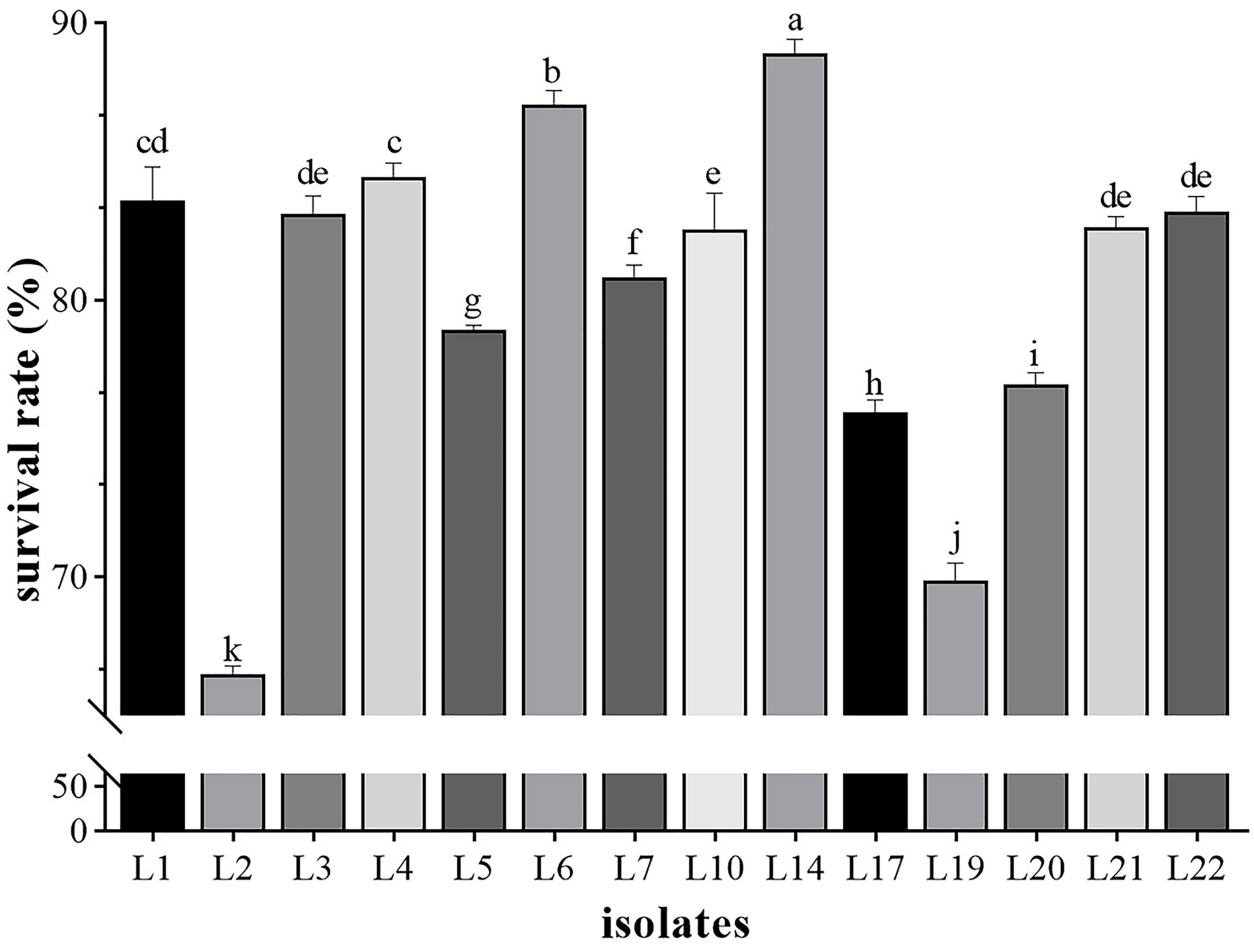
The results of phenol tolerance of 14 LAB isolates. Results are expressed as mean ± SD of triplicate tests. ^a-k^ Values in a column with different superscript letters are significantly different (Waller-Duncan, *p* < 0.05).

The influence of phenol on the growth of these 14 LAB isolates is shown in Figure 2, in which L14 showed the highest phenol tolerance (88.91%), followed by L6 (87.03%), L4 (84.45%), and L1 (83.57%). Except for L2 (66.50%) and L19 (69.86%), all isolates tolerated 0.4% phenol (>70%).

### Adhesion activity

Different auto-aggregation abilities were revealed for the selected LAB isolates at the 3rd h, 6th h, and 24th h from the beginning of co-culture, and the results are shown in Table 4. In general, the auto-aggregation effect of the identified LAB isolates showed a time-dependent manner. At the 3 h point, L20 showed the highest auto-aggregation ability (76.32%), followed by L14 (62.50%), L7 (49.28%), and L17 (40.73%), while the other tested isolates showed lower auto-aggregation abilities between 1.81 and 27.87%. At 6 h point, the isolates with high auto-aggregation effect were L20 (86.37%), L14 (79.61%), L7 (77.64%), L4 (58.62%), and L19 (53.49%). Whereas, at the 24 h point, the auto-aggregation effect of seven isolates (L4, L14, L20, L7, L5, L19, and L17) exceeded 80%, and the others (except L10) also showed high auto-aggregation effects (53.26–65.31%).

**Table 4:** Auto-aggregation abilities of potential probiotic LABs from cow milk.^1^

Strain	Auto-aggregation (%)		
	3 h	6 h	24 h
L1	13.00 ± 2.75^gh^	26.98 ± 1.50^f^	65.31 ± 5.70^c^
L2	10.10 ± 2.02^h^	18.07 ± 1.90^h^	53.26 ± 10.03^de^
L3	15.80 ± 2.54^g^	23.31 ± 0.49^g^	58.70 ± 8.04^d^
L4	27.39 ± 5.64^e^	58.62 ± 1.86^c^	91.62 ± 0.47^a^
L5	22.36 ± 1.46^f^	38.68 ± 1.47^e^	88.51 ± 0.91^ab^
L6	13.28 ± 1.92^gh^	23.62 ± 1.07^g^	55.96 ± 4.39^d^
L7	49.28 ± 1.42^c^	77.64 ± 1.26^b^	88.88 ± 0.58^ab^
L10	1.81 ± 0.72^i^	13.47 ± 3.16^i^	47.83 ± 0.67^e^
L14	62.50 ± 0.51^b^	79.61 ± 0.98^b^	90.33 ± 1.26^a^
L17	40.73 ± 2.94^d^	39.44 ± 0.81^e^	83.91 ± 0.34^b^
L19	27.87 ± 0.92^e^	53.49 ± 2.72^d^	87.76 ± 0.31^ab^
L20	76.32 ± 2.27^a^	86.37 ± 0.37^a^	89.63 ± 0.64^ab^
L21	13.79 ± 0.42^gh^	26.18 ± 1.22^f^	57.19 ± 0.82^d^
L22	14.83 ± 1.64^g^	25.01 ± 2.20^fg^	57.40 ± 1.38^d^

1 *Data are mean ± SD from triplicate experiments;*

^a-i^*Values in a column with different superscript letters are significantly different (Waller-Duncan, p < 0.05)*.

**Figure 3:**
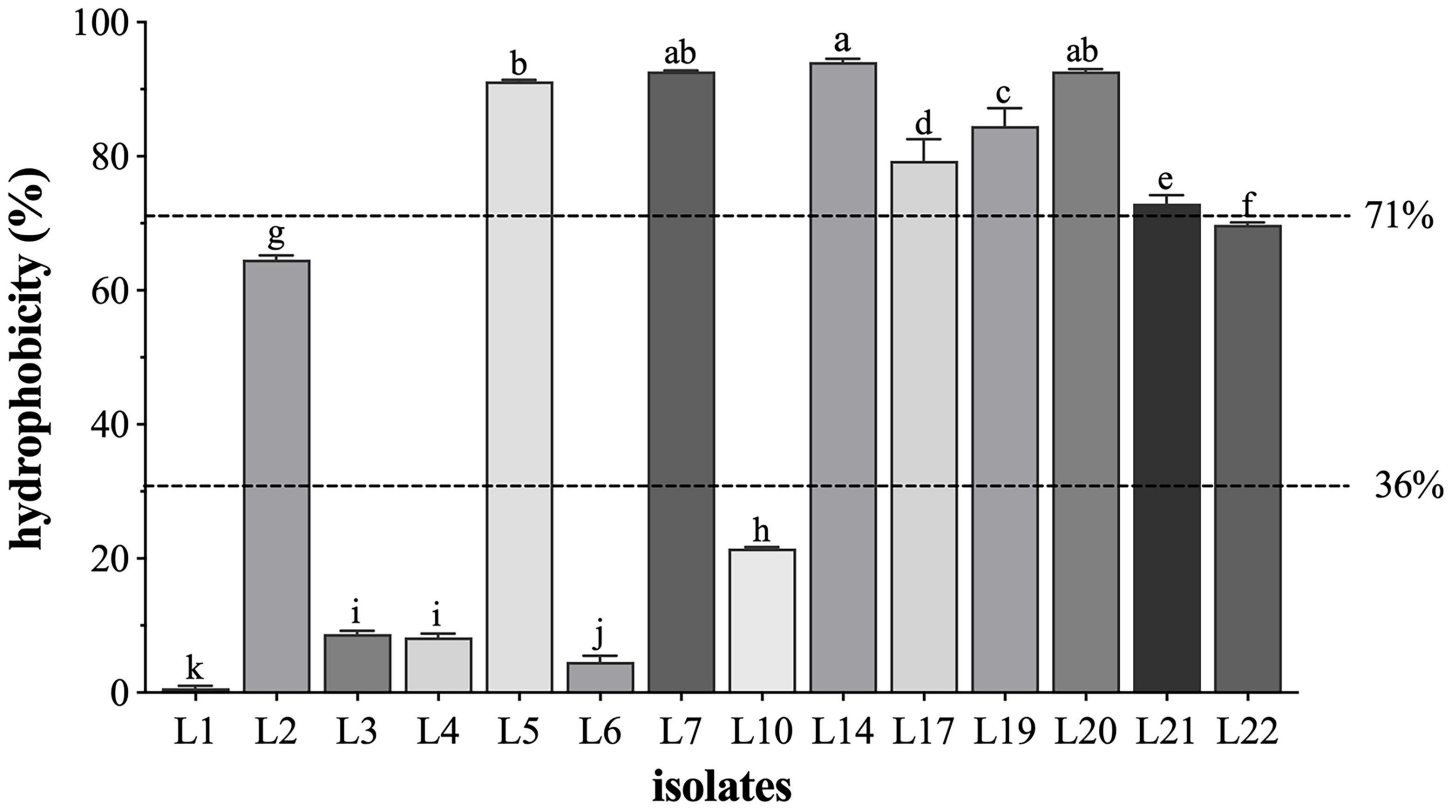
The cell surface hydrophobicity of 14 LAB isolates. Results are expressed as mean ± SD of triplicate tests. ^a-k^ Values in a column with different superscript letters are significantly different (Waller-Duncan, *p* < 0.05). The dotted lines represent the threshold to distinguish the surface hydrophobicity (high, medium, and low) of the tested isolates.

The results of the cell surface hydrophobicity of the identified LAB isolates are shown in Figure 3. In general, most of the identified isolates showed high (71 ~ 100%) or medium (36 ~ 70%) cell surface hydrophobicity (the classification standard referred to Ocana et al. (Ocaña et al., 1999)). The hydrophobicity was highest in L14 (94.08%), followed by L20 (92.67%), L7 (92.70%), L5 (91.21%), L19 (84.52%), L17 (79.32%), and L21 (72.95%), and L22 (69.81%) and L2 (64.58%) showed the medium hydrophobic.

#### Safety analysis

The hemolytic activity test results showed that these 14 isolates were non-hemolytic (Supplementary Figure 1). The susceptibility profile of all these 14 isolates to 13 commonly used antibiotics was assessed, and the results are shown in Table 5. The resistance rates (includes resistance and intermediate) were 0% (0/14) to penicillin G), 64.28% (9/14) to ceftriaxone, 100% (14/14) to vancomycin, 14.29% (2/14) to chloramphenicol, 100% (14/14) to gentamicin, 64.28% (9/14) to erythromycin, 85.71% (12/12) to tetracycline, 57.14% (8/14) to rifampicin, 7.14% (1/14) to ampicillin, 100% (14/14) to amikacin, 7.14% (1/14) to amoxicillin, 28.57% (4/14) to clarithromycin, and 100% (14/14) to streptomycin, respectively. L20 showed the highest sensitive rate (76.92%), L3, L5, L7, L14, L19, and L21 showed a higher sensitive rate (69.23%) to these 13 antibiotics.

**Table 5:** Antibiotic susceptibility results of LAB isolates from milk.

Strain	Antibiotic susceptibility*													Sensitive rate (S + I, %)
	P	CRO	VA	C	CN	E	TE	RD	AMP	AK	AML	CLR	S	
L1	S	R	R	S	R	S	I	S	S	R	S	S	R	61.54
L2	S	S	I	I	R	R	S	R	R	R	R	R	R	38.46
L3	S	I	R	S	R	I	I	S	S	R	S	S	R	69.23
L4	S	S	R	S	R	I	I	R	S	R	S	S	R	61.54
L5	S	I	R	S	R	S	I	S	S	R	S	I	R	69.23
L6	S	R	R	S	R	I	I	S	S	R	S	S	R	61.54
L7	S	I	R	S	R	I	I	I	S	R	S	S	R	69.23
L10	S	R	R	S	R	I	I	I	S	R	S	S	R	61.54
L14	S	I	R	S	R	I	I	S	S	R	S	I	R	69.23
L17	S	S	R	I	R	R	I	R	S	R	S	I	R	53.85
L19	S	S	R	S	R	S	S	I	S	R	S	S	R	69.23
L20	S	S	R	S	I	S	I	S	S	R	S	S	R	76.92
L21	S	I	R	S	R	I	I	I	S	R	S	S	R	69.23
L22	S	R	R	S	R	S	I	I	S	R	S	S	R	61.54

*P*, penicillin G; CRO, ceftriaxone, VA, vancomycin; C, chloramphenicol; CN, gentamicin; E, erythromycin; TE, tetracycline; RD, rifampicin; AMP, ampicillin; AK, amikacin; AML, amoxicillin; CLR, clarithromycin; and S, streptomycin. *R, Resistance; S, Sensitive; and I, Intermediate.

### Growth curve

**Figure 4:**
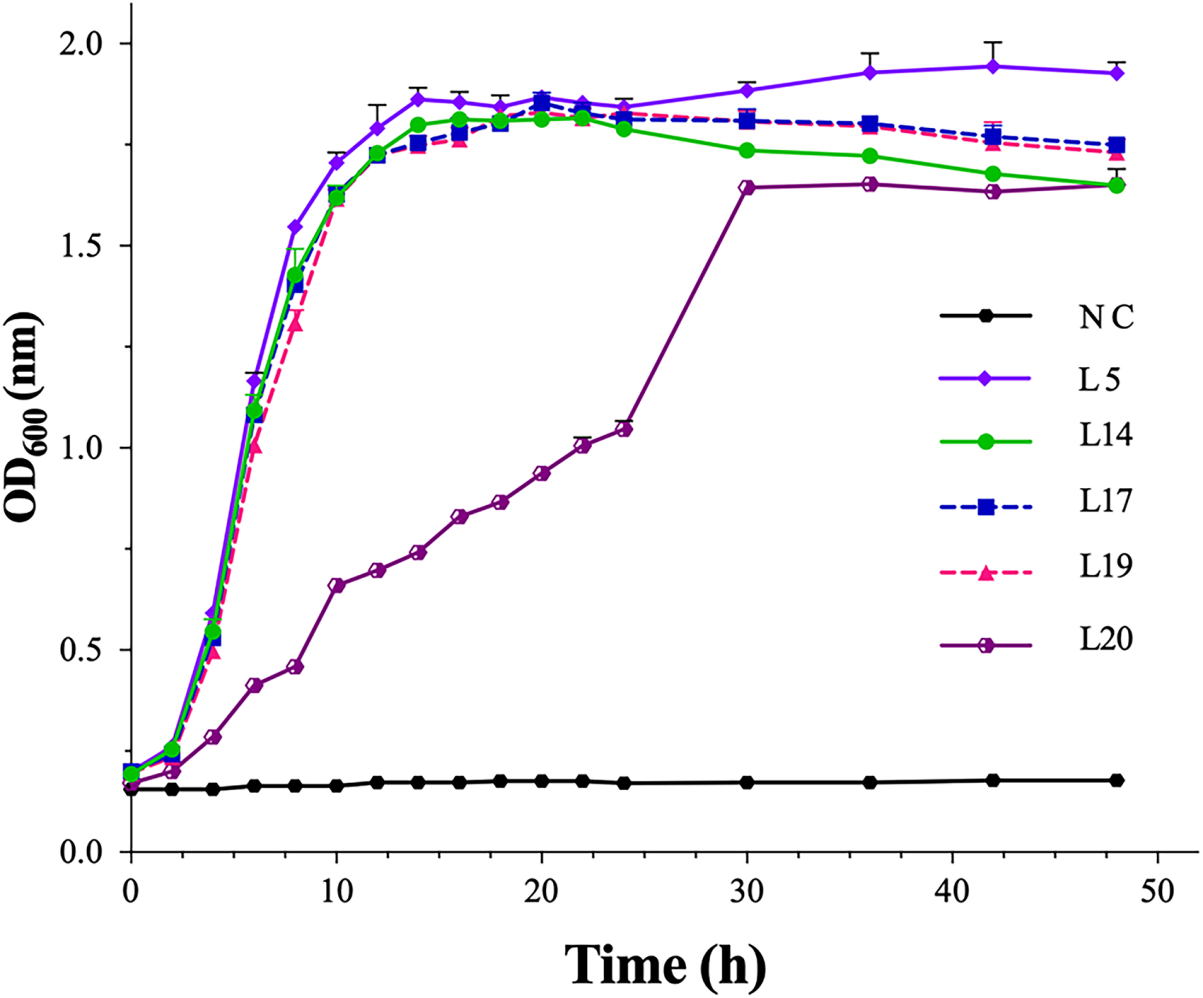
The growth curves of the five most probiotic potential isolates. The optical density at 600 nm (OD_600_ nm) of the cultures. NC, sterile MRS medium. Error bars refer to the SD of the 3 replicates of each assay.

Based on the results of the probiotic property test and safety analysis, the growth characteristics of the five most probiotic potential isolates, L5, L14, L17, L19, and L20, were measured, and their growth curves are shown in Figure 4. Except for L20, the other four isolates entered the stationary phase at the 14th h and lasted until the 48^th^ h, indicating their good growth performance. L20 reached the stationary phase at the 30th h. L5 showed the best growth performance, while L20 showed the worst.

## Discussion

The high nutrient content of raw milk provides multiple kinds of bacteria with favorable circumstances (Quigley et al., 2013), and the microbiota profiles from different farms are different (Hornik et al., 2021). LABs, a group of bacteria that ferment lactose to lactate, are a dominant population in cow milk before pasteurization. Hence, raw cow milk was considered an important source of LABs. The raw milk from this farm with 50 years of history had never been sampled for LAB isolation, and we obtained five isolates with promising probiotic potential.

Antipathogenic activity and safety characteristics were considered the most important properties of probiotic LABs (Araya et al., 2002). Hence, we detected the antagonistic activity of these 26 isolates against intestinal pathogens. Four standard common pathogenic strains, ETEC (Fleckenstein and Kuhlmann, 2019), *S. aureus* (Cheung et al., 2021), *Salmonella* (Knodler and Elfenbein, 2019), and *P. aeruginosa* (Bachta et al., 2020), were used as target pathogens for antagonistic activity assay, and 14 LAB strains were found to inhibit the growth of all these pathogenic strains (Table 1). In the present study, the inhibition zones against *S. aureus* by the 14 obtained LAB strains were (> 20 mm) much wider than those isolated from human milk (Heikkila and Saris, 2003; Makete et al., 2017; Pellegrino et al., 2019). The strong *S. aureus* antagonistic activities of the LAB strains indicated that the cows might suffer from *S. aureus-*induced mastitis in the past (Pellegrino et al., 2019). Moderate inhibition zones against *P. aeruginosa*, ETEC and *Salmonella* were recorded, indicating a considerable antagonistic activity of these 14 LAB strains, most of which (L1, L5, L10, L14, L17, L19, L20, L21, and L22) showed relative higher antagonistic activities against the target pathogenic strains (Table 1). Intriguing, the antimicrobial ability of LAB strains is mainly produced by the secreted compounds (such as organic acids, hydrogen peroxide, and bacteriocin), and the special microenvironment in the GIT (enzymes, adverse pH, and mild heat shock, et al.) further enhances the antimicrobial potency of these compounds (Gänzle et al., 1999), indicating that they might exhibit higher antimicrobial abilities if they are orally taken. The co-aggregative ability is another antagonistic probiotic activity which boosts pathogen agglomeration with probiotic cells and facilitates its elimination through feces (De Melo Pereira et al., 2018). In our experiment, L5, L7, L14, L19, L20, and L22 showed higher co-aggregation abilities (up to 90%) against all target pathogenic strains (Table 2), indicating that these LAB isolates can easily agglomerate with enteric pathogens and eliminate them. These results proved the considerable antipathogenic activity of these 14 LAB isolates.

After oral administration, the probiotics will have to face all the antimicrobial factors in the stomach (pepsin, gastric acid, and low pH) and intestines (bile salts, trypsin, and high pH), as well as mild heat stimulus caused by the internal body temperature (approximate 37.5°C), which forces the probiotics must have acid and bile tolerance or other exclusion mechanisms to survive in the gut (De Melo Pereira et al., 2018). Previous studies revealed that the tolerant abilities of LAB strains are strain-specific (Liu et al., 2020; Reuben et al., 2020). Similarly, in the present study, the 14 selected LAB isolates showed varying survival rates (33 ~ 84%) after 3 h of low pH gastric acid-containing pepsin, followed by 4 h of high pH intestinal juice-containing ox-bile salts and trypsin, indicating their heterogeneous tolerance for bile salts and acidic gastric (Table 3). It is important to note that the survival rates of our LAB isolates in the simulated gastric acid and intestinal juice were up to 99.69 and 84.94%, respectively, which are higher than those of LABs in recent reports (Liu et al., 2020; Reuben et al., 2020). This high tolerance allows them to survive longer, colonize in GIT environments, and keep effective when administered (Prasad et al., 1998). Phenol, a kind of GIT secreted toxic metabolite that might inhibit the growth of probiotics, is another challenge that poses stresses to the ingested probiotics (Barbour and Vincent, 1950), which means the probiotic candidates must be able to endure the bacteriostatic action of phenol to exert the optimal beneficial effects on the hosts. In the present study, the 14 LAB isolates exhibited varying degrees of tolerance (66 ~ 88%) for 0.4% phenol at 37°C (Figure 2), which are also higher than those in the previous report (Shehata et al., 2016; Reuben et al., 2020). These results indicated that the six LAB isolates, L5, L7, L14, L17, L21, and L22, are able to survive in the GIT, which has a promising probiotic potential.

The ability of adhesion to intestinal cells is considered an essential criterion for probiotic selection (Collado et al., 2006; De Melo Pereira et al., 2018). The adhesion process to epithelial cells is complex, involves the membranes of both microbial and human cells, and depends on the chemical and physicochemical composition of the strain cell’s surface, affected by the strain extracellular components and their surrounding composition (Duary et al., 2011). Even though we did not directly explore the adhesion abilities of the 14 LAB isolates to epithelial cells in the present study, the auto-aggregation capacity and hydrophobic properties were evaluated to assess the adhesion abilities indirectly. The auto-aggregation ability ensures that the strains reach a high cell density in the gut, contributing to the adhesion mechanism. Previous studies reported that the LAB strains isolated from raw milk showed no or low auto-aggregation (Espeche et al., 2009, 2012). However, in the present study, we showed that the auto-aggregation at the 3^rd^ h was moderate (< 50%), but they were up to 89% at the 24^th^ h, which is much higher than those in the previous reports, indicating that the auto-aggregation of these LAB isolates increases with time. At the same time, hydrophobicity allows increased interaction between probiotics and host epithelial cells (De Melo Pereira et al., 2018). Hence, we also evaluated the hydrophobicity of the selected LAB strains in our study, and the results showed that L5, L7, L14, L17, L19, L20, and L21 possessed high hydrophobicity (up to 90%; Figure 3), much higher (80%) than those in a previous study (Sirichokchatchawan et al., 2018). Combined with the autoaggregation assessment results, it was concluded that L5, L7, L14, L17, L19, and L20 had high adhesion activities and were easily adherent to intestinal cells to exert their probiotic effects.

The first step to assessing the safety of probiotics is the identification (Yadav and Shukla, 2017), and strain-level identification was highlighted by the Natural Health Products Regulations (NHPR) for probiotic safety establishment in human health (Coeuret et al., 2004). In the present study, the 26 obtained LAB isolates were identified at the species level based on the biochemical and 16S rDNA sequencing results, and 13 of them were identified as *Lactiplantibacillus plantarum*, an ideal probiotic in the food industry (Seddik et al., 2017). Notably, these isolates showed huge variations in probiotic properties, although all of them belong to *Lactiplantibacillus plantarum*, indicating that they have different gene sequences in their non-16 S rDNA gene regions, which needs to be further confirmed by genomic comparison. However, biochemical and genetic analyses are insufficient to compare probiotic bacteria at the strain level (De Melo Pereira et al., 2018). In addition, it was expected that probiotic candidates must not lyse red blood cells when ingested by humans or animals. Hence, we also assessed the hemolytic activities of the 14 LAB isolates. Similar to previous studies (Santini et al., 2010; Reuben et al., 2020), none of them were hemolytic (Supplementary Figure 1). Furthermore, in this study, we detected the antibiotic susceptibilities of the selected 14 LAB isolates against 13 commonly used antimicrobials, and the results showed that the LAB isolates all had high sensitivity (> 60%) except for L2 (Table 5). Intriguing, all isolates were resistant to streptomycin, amikacin, gentamicin (except for 20), and vancomycin (except for L2), which is consistent with previous reports (Liasi et al., 2009; Reuben et al., 2020). It is supposed that the resistance against these four antimicrobials might be associated with LABs’ innate resistance caused by the membrane’s impermeability, probably through a resistance efflux mechanism (Liasi et al., 2009). On the other hand, the strain-specific molecular mechanism of this intrinsic antimicrobial resistance by these LAB isolates needs further in-depth study because this inherent resistance might promote both preventive and therapeutic outcomes when the probiotics are administered together with antibiotics (Jose et al., 2015). Fortunately, all 14 isolates were sensitive to penicillin G, tetracycline, ampicillin (except for L2), amoxicillin (except for L2), and clarithromycin (except for L2), the five most commonly used antibiotics in humans. Combined with the results of biochemical and genotypic identification, hemolytic activities, and antibiotic susceptibilities, it could be concluded that these selected LAB isolates were safe for use except for L2.

Taken together with the results of these *in vitro* probiotic evaluation tests, five *Lactiplantibacillus plantarum* isolates, L5, L14, L17, L19, and L20, showed promising probiotic potential and were considered the probiotic candidates. These five isolates were isolated from distinct samples and showed varying probiotic properties, indicating their different gene sequences and biological features, which need further study. Mounts of probiotic cells and byproducts can be obtained by the selected LAB strains if they are cultured under a specific, controlled condition with ample nutrient supply (Kuznetsov et al., 2017). In the present study, the growth performance of the five selected probiotic isolates under the common condition (in MRS broth, at 37°C, pH = 6.2), different from the conditions in the gut or under industrial production, was also evaluated (Figure 4). Similarly, they showed different growth performances under the same condition. Interestingly, L20 showed an atypical growth curve, indicating that this condition might not be optimal. Hence, further studies need to be conducted to evaluate the growth performance of the selected isolates under different situations. It is important to note that these *in vitro* evaluations are not adequate to claim these LAB strains as probiotics, and further *in vivo* and clinical trials need to be carried out. In addition, even if the probiotic performance of these five candidates was proved to be relatively better by animal studies than those reported in some other manuscripts, it also needs to be compared with those existing commercialized probiotics to confirm their better application potential in industrialization. Recently, Clustered Regularly Interspaced Short Palindromic Repeats (CRISPR)-Cas 9, a newly developed and very promising gene-editing tool, might enable us to efficiently edit the gene sequences of probiotic candidates to obtain ideal probiotics by knocking down their virulence genes or resistant genes with horizontal transfer capabilities, and overexpressing probiotic effect related genes (Sirichokchatchawan et al., 2018; Goh and Barrangou, 2019).

## Conclusion

Cow milk contains massive bacteria and is important for probiotics isolation, especially LABs. In the present study, we obtained 26 LABs from raw milk collected from a farm whose milk had never been used for LAB isolation. The isolated LAB isolates were identified at the species level by biochemical and 16S rDNA sequencing methods. The probiotic properties of these 26 LABs isolates were screened *via* several *in vitro* experiments, including antagonistic activity, co-aggregation ability with pathogens, tolerance for simulated GIT conditions and phenol, autoaggregation activity, cell surface hydrophobicity, hemolytic activity, and antibiotic susceptibility. Furthermore, four *Lactiplantibacillus plantarum* strains, named L5, L14, L17, and L19, showed comprehensive probiotic properties and considerable growth performance, which need to be further confirmed *via in vivo* studies and clinical trials. This study revealed the probiotic properties of LAB isolates collected from raw milk of a local feedlot and provided five LAB isolates with promising probiotic potential for application in the food and pharmaceutical industries.

## Data availability statement

The datasets presented in this study can be found in online repositories. The names of the repository/repositories and accession number(s) can be found in the article/Supplementary material.

## Author contributions

GP and WZ: conceive and design this study. WZ and SL: sample collection and main experiments. WZ and JY: data analysis and writing—original draft preparation. LD, ZZ, and GP: supervision and writing—reviewing and editing. All authors reviewed the manuscript. All authors contributed to the article and approved the submitted version.

## Funding

This study was financially supported by the National Science and Technology Department’s “13th Five-Year” Special Subproject of China (No. 2016YFD0501009).

## Conflict of interest

The authors declare that the research was conducted in the absence of any commercial or financial relationships that could be construed as a potential conflict of interest.

## Publisher’s note

All claims expressed in this article are solely those of the authors and do not necessarily represent those of their affiliated organizations, or those of the publisher, the editors and the reviewers. Any product that may be evaluated in this article, or claim that may be made by its manufacturer, is not guaranteed or endorsed by the publisher.

## Footnotes

^1^ https://www.ncbi.nlm.nih.gov/genbank/

^2^ http://blast.ncbi.nlm.nih.gov/Blast.cgi

## Supplementary material

The Supplementary materials for this article can be found online at: https://www.frontiersin.org/articles/10.3389/fmicb.2022.918774/full#supplementary-material

SUPPLEMENTARY FIGURE 1 The results of the hemolytic test of 14 selected isolates.
