# Supplementary figures and images for: Screening and evaluation of lactic acid bacteria with probiotic potential from local Holstein raw milk
Source: Front Microbiol. 2022 Aug 1;13:918774. doi: 10.3389/fmicb.2022.918774 (PMC9377552; doi:10.3389/fmicb.2022.918774)

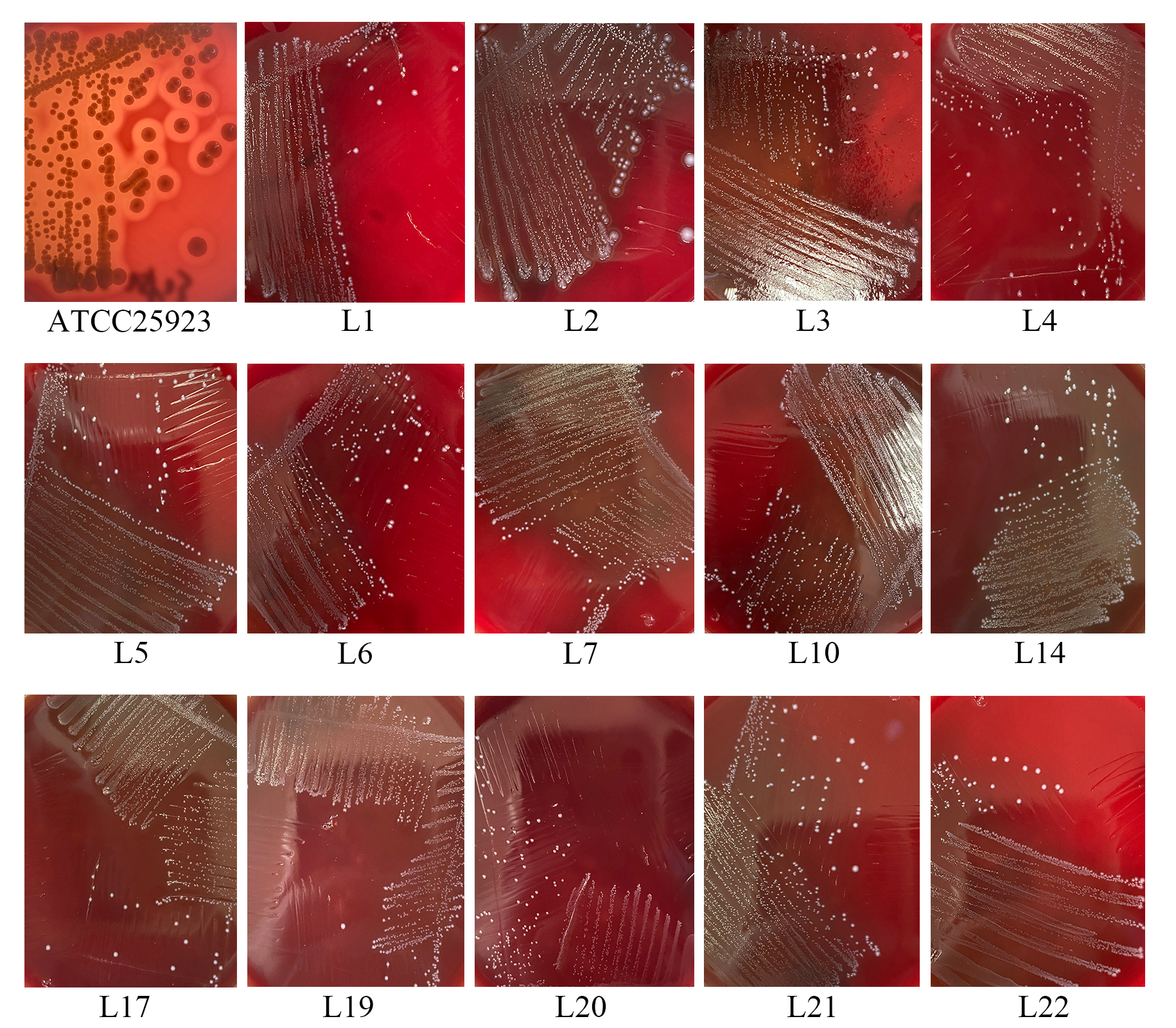

Supplement: SUPPLEMENTARY FIGURE 1 — The results of the hemolytic test of 14 selected isolates. [file Image_1.tif]
