# Supplementary material for: Screening and evaluation of lactic acid bacteria with probiotic potential from local Holstein raw milk
Source: Front Microbiol. 2022 Aug 1;13:918774. doi: 10.3389/fmicb.2022.918774 (PMC9377552; doi:10.3389/fmicb.2022.918774)
Supplement: Supplementary file 2 [file Table_1.docx]

Supplementary Table 1: The biochemical characteristics of the 26 LAB isolates.

| Tests | Isolates | | | | | | | | | | | | | | | | | | | | | | | | | | |
| --- | --- | --- | --- | --- | --- | --- | --- | --- | --- | --- | --- | --- | --- | --- | --- | --- | --- | --- | --- | --- | --- | --- | --- | --- | --- | --- | --- |
|  | L1 | L2 | L3 | L4 | L5 | L6 | L7 | L8 | L9 | L10 | L11 | L12 | L13 | L14 | L15 | L16 | L17 | L18 | L19 | L20 | L21 | L22 | L23 | L24 | L25 | L26 |  |
| Gram-staining | + | + | + | + | + | + | + | + | + | + | + | + | + | + | + | + | + | + | + | + | + | + | + | + | + | + |  |
| Catalase reaction | - | - | - | - | - | - | - | - | - | - | - | - | - | - | - | - | - | - | - | - | - | - | - | - | - | - |  |
| Gelatin | - | - | - | - | - | - | - | - | - | - | - | - | - | - | - | - | - | - | - | - | - | - | - | - | - | - |  |
| Nitrate reduction | - | - | - | - | - | - | - | - | - | - | - | - | - | - | - | - | - | - | - | - | - | - | - | - | - | - |  |
| H2S production | - | - | - | - | - | - | - | - | - | - | - | - | - | - | - | - | - | - | - | - | - | - | - | - | - | - |  |
| Sucrose | + | + | + | - | + | + | + | + | - | + | - | + | + | + | + | - | - | - | - | - | + | + | + | + | + | - |  |
| Xylose | + | + | + | - | + | + | + | + | - | + | + | - | + | + | - | + | - | + | - | + | + | + | + | - | - | + |  |
| Lactose | + | + | + | + | + | + | + | + | + | + | + | - | + | + | + | + | + | + | + | - | + | + | + | + | + | + |  |
| Cellobiose | + | + | + | + | + | + | + | + | + | + | + | + | + | + | + | + | + | - | + | - | + | + | + | - | - | + |  |
| Esculin | + | + | + | + | + | + | + | + | + | + | + | + | + | + | + | + | + | + | + | + | + | + | - | - | + | - |  |
| Maltose | + | - | + | + | + | + | + | + | + | + | - | + | - | + | + | + | + | + | + | + | + | + | + | + | + | + |  |
| Sorbitol | + | - | + | + | + | + | + | - | - | + | - | - | - | + | - | - | + | - | + | + | + | + | - | - | + | + |  |
| Mannitol | + | - | + | + | + | + | + | - | - | + | - | + | - | + | + | - | + | - | + | + | + | + | - | - | - | + |  |
| Salicin | + | + | + | + | + | + | + | + | + | + | + | - | + | + | + | - | + | - | + | + | + | + | - | - | - | + |  |
| Fructose | + | - | + | + | + | + | + | + | + | + | - | + | - | + | + | + | + | + | + | + | + | + | + | + | + | + |  |
| Raffinose | + | - | + | - | + | + | + | + | - | + | - | - | - | + | - | - | - | - | - | - | + | + | + | + | - | - |  |

Note: +, positive; -, negative.
